# A Novel Imprinted Gene *NUWA* Controls Mitochondrial Function in Early Seed Development in *Arabidopsis*

**DOI:** 10.1371/journal.pgen.1006553

**Published:** 2017-01-17

**Authors:** Shan He, Yan Sun, Qian Yang, Xiangyu Zhang, Qingpei Huang, Peng Zhao, Mengxiang Sun, Jingjing Liu, Weiqiang Qian, Genji Qin, Hongya Gu, Li-Jia Qu

**Affiliations:** 1 State Key Laboratory of Protein and Plant Gene Research, Peking-Tsinghua Center for Life Sciences, School of Life Sciences, Peking University, Beijing, China; 2 Department of Cell and Development Biology, College of Life Science, State Key Laboratory of Hybrid Rice, Wuhan University, Wuhan, China; 3 The National Plant Gene Research Center (Beijing), Beijing, China; The University of North Carolina at Chapel Hill, UNITED STATES

## Abstract

Imprinted genes display biased expression of paternal and maternal alleles and are only found in mammals and flowering plants. Compared to several hundred imprinted genes that are functionally characterized in mammals, very few imprinted genes were confirmed in plants and even fewer of them have been functionally investigated. Here, we report a new imprinted gene, *NUWA*, in plants. *NUWA* is an essential gene, because loss of its function resulted in reduced transmission through the female gametophyte and defective cell/nuclear proliferation in early *Arabidopsis* embryo and endosperm. *NUWA* is a maternally expressed imprinted gene, as only the maternal allele of *NUWA* is transcribed and translated from gametogenesis to the 16-cell globular embryo stage after fertilization, and the *de novo* transcription of the maternal allele of *NUWA* starts from the zygote stage. Different from other identified plant imprinted genes whose encoded proteins are mostly localized to the nucleus, the NUWA protein was localized to the mitochondria and was essential for mitochondria function. Our work uncovers a novel imprinted gene of a previously unidentified type, namely, a maternal-specific expressed nuclear gene with its encoded protein localizing to and controlling the function of the maternally inherited mitochondria. This reveals a unique mechanism of maternal control of the mitochondria and adds an extra layer of complexity to the regulation of nucleus-organelle coordination during early plant development.

## Author Summary

Imprinted genes are only found in mammals and flowering plants, and they express with parent-of-origin-specific patterns. Unlike in mammals, only a few imprinted genes was identified and functionally characterized in plants, and almost all of them encode nuclear proteins. Here, we identified *NUWA* as a new type of imprinted gene in *Arabidopsis*. *NUWA* has maternal-specific expression and controls the early seed development in *Arabidopsis*, with its encoded protein localizing to and functioning in the maternally inherited mitochondria. The discovery of *NUWA* reveals a unique mechanism of maternal control of mitochondrial function and adds an extra layer of complexity to nucleus-organelle coordination. It further indicates that, similar to mammals, imprinted genes in plants are also involved in various subcellular biological processes.

## Introduction

Genomic imprinting is a phenomenon in which somatic cells express some genes only from the maternal or paternal chromosome [1]. Genes with parent-of-origin-specific allele-biased expression patterns are called imprinted genes [2,3]. Imprinted genes have only been discovered and confirmed in placental mammals and flowering plants and are thought to have evolved independently through convergent evolution in these two groups [4,5]. Imprinting is regulated epigenetically and plays important roles in mediating complex traits in both mammals and plants [6–8]. In mammals, imprinted genes are essential not only in embryonic development and placental development of the fetus but also in sensory function and behavior in adults [9–11]. Loss-of-function of these genes always results in severe disease [12,13]. In plants, imprinted genes are also involved in early development, and the loss-of-function of these genes can lead to seed-lethal phenotypes [2,3].

In comparison with the hundreds of functional imprinted genes identified in mammals, confirmed imprinted genes in plants are rare, and many of them do not have obvious functions, because no phenotypes were observed in the loss-of-function mutants [1,13–17]. In addition, products of the imprinted genes in mammals have a variety of subcellular localizations and numerous molecular functions [18], whereas in plants, products of most of the confirmed imprinted genes are localized in the nucleus, and many are presumed to function in chromatin remodeling [19–28]. Although high-throughput analyses revealed many putative imprinted genes in *Arabidopsis* [29,30], maize [31] and rice [32,33], most of them have not been confirmed. It is not known whether the imprinted genes in plants are important in many aspects of subcellular and biological processes, as in mammals, and the driving force of the convergent evolution of genomic imprinting therefore remains a mystery.

In this study, we have identified a novel maternally expressed imprinted gene *NUWA* in *Arabidopsis*, which is named after the well-known goddess in Chinese ancient mythology who created humans by molding figures from earth. *NUWA* is an essential gene. Loss of *NUWA* function resulted in defects in cell/nuclear proliferation in early embryogenesis and endosperm development. Moreover, *NUWA* is a maternally expressed imprinted gene, because *de novo* transcription of maternal allele-specific *NUWA* was detected in the embryo sac after fertilization. Different from those previously-identified plant imprinted genes whose products were localized to the nucleus, the NUWA protein was targeted to the mitochondria and was essential for development and proper function of the mitochondria. These results indicate that *NUWA* is a new type of imprinted gene that maternally controls early embryo and endosperm development through regulating the function of the maternally inherited mitochondria. The discovery of *NUWA* also reveals a new aspect of subcellular and metabolic processes in which plant imprinted genes are involved.

## Results

### *nuwa* is a mutant with defects in early embryogenesis and endosperm development

**Fig 1:**
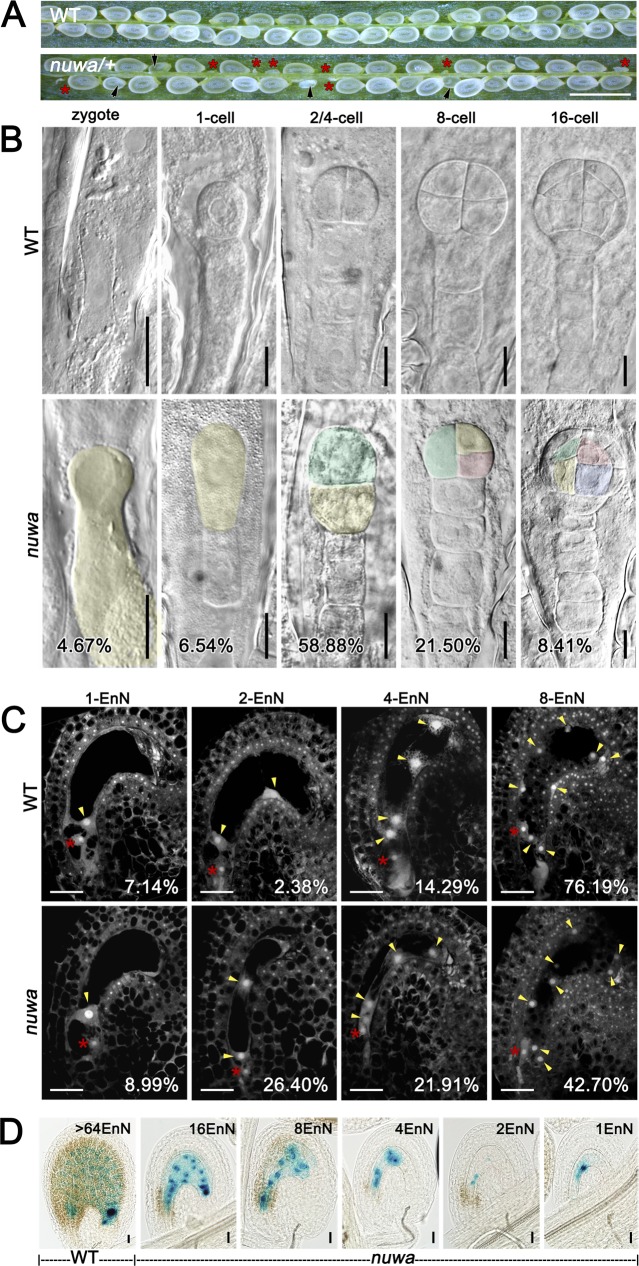
*nuwa* is a mutant with early embryo and endosperm developmental defects. (A) Seed set of wild type and *nuwa/+* siliques. Red stars and black arrows indicate earlier and later aborted seeds, respectively. Bar = 1 mm. (B) Early-stage embryos observed in wild type plant (upper row) and in *nuwa/+* (lower row). Cells with abnormal morphology are marked by pseudo-colors. Percentages of aborted embryos are shown. Bars = 10 μm. (C) Endosperm nuclei development in 26 HAP ovules of wild type (upper row) and selfed *nuwa/+* plants (lower row). Percentages of endosperm are shown. Red stars indicate embryo nuclei; yellow arrowheads mark endosperm nuclei. EnN, endosperm nuclei. Bars = 20 μm. (D) Ovules of *nuwa/+* with the endosperm nuclei marked with FIS2-GUS. EnN, endosperm nuclei. Bars = 20 μm.

The mutant *nuwa/+* was obtained from genetic screening of a mutant collection with a Basta/Phosphinothricin (PPT)-resistant gene in the T-DNA [34]. In *nuwa/+* siliques, 37.5% of seeds aborted very early; 1.6% of seeds aborted somewhat later (n = 6685) (Fig 1A). In selfed *nuwa/+* progeny, the PPT-resistant (PPT^r^) to PPT-sensitive (PPT^s^) ratio was approximately 1.33:1 (n = 4387). No *nuwa/-* homozygous mutant plants were acquired.

When we pollinated *nuwa/+* with pollen of a fertilization indicator line FAC1-GUS, whose GUS signal appears 3 hours after fertilization in embryo sacs [35], the percentage of ovules with GUS signals was indistinguishable between wild type and *nuwa/+* (|u| = 0.248 < u_0.05_ = 1.96, *P* > 0.05) (S1A to S1C Fig), suggesting that all of the defective female gametophytes of *nuwa/+* could be fertilized. Therefore, we hypothesized that the reduction in the seed setting rate in *nuwa/+* probably resulted from a defect in the embryo and/or endosperm after fertilization.

Using differential interference contrast (DIC) microscopy, we found that mutant embryos of *nuwa/+* were arrested before the 16-cell stage. Most mutant embryos were arrested at the first three rounds of cell division, and some displayed abnormal cell shapes and irregular cell division patterns (Fig 1B). This result suggested that the mutant embryos were arrested at very early developmental stage, with cell division defects. Then, we analyzed endosperm development using confocal laser scanning microscopy. Only 42.7% of *nuwa/+* ovules at 26 hours after pollination (HAP) were at the 8-endosperm-nuclei stage, which was significantly lower than the percentage in wild-type ovules (78.6%) (|u| = 3.91 > u_0.01_ = 2.58, *P* < 0.01) (Fig 1C). The percentage of ovules at 4-endosperm-nuclei stage and 2-endosperm-nuclei stage in *nuwa* were significantly higher than those in wild type (Fig 1C), indicating that the endosperm nuclei proliferations were slower in the mutant. After we crossed *nuwa/+* with FIS2-GUS [36], we found that fewer spherical GUS signals indicating endosperm nuclei were observed in mutant ovules compared with normal ovules (Fig 1D, S1D to S1I Fig), further supporting the notion that endosperm nucleus proliferation is delayed in mutant ovules. The facts that both embryo and endosperm of *nuwa*/+ developed slowly after fertilization and were arrested at early stages demonstrate that *NUWA* is an essential gene.

### *NUWA* encodes a conserved protein which is highly expressed in reproductive organs

**Fig 2:**
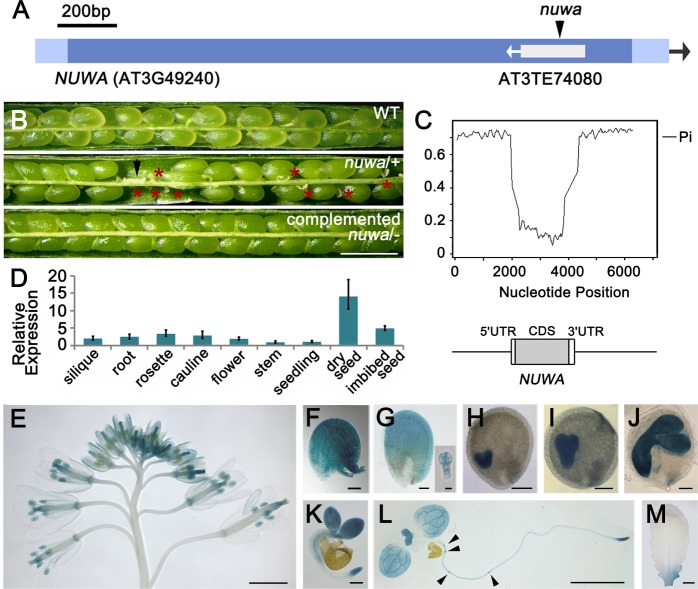
*NUWA* is mainly expressed in reproductive organs. (A) The T-DNA insertion sites in *nuwa/+*. (B) Siliques of wild type (upper), *nuwa/+* (middle) and complemented *nuwa/-* (lower). Red stars and the black arrow indicate early and late aborted seeds, respectively. Bar = 1 mm. (C) Nucleotide diversity in the genomic region centered on *NUWA* among 5 Brassicaceae species, *i*.*e*., *A*. *thaliana*, *A*. *lyrata*, *C*. *rubella*, *B*. *rapa* and *E*. *salsugineum*. (D) Real-time qPCR analysis of *NUWA* in various tissues. Error bars, mean ± SD. (E-M) GUS staining patterns in inflorescence (E), ovule before fertilization (F), ovule at 2–4 cell embryo stage (G), ovules at heart (H), torpedo (I), walking-stick (J) embryo stages, germinated seeds (K), 10-day-old seedlings (L) and rosette leaf (M) in *pNUWA*:*NUWA*-GFP-GUS transgenic plants. Arrowheads indicate lateral root primordia. Bars = 2 mm (E, L, M), 50 μm (F, G), 100 μm (H-J), 200 μm (M) or 10 μm (small figure in G).

Using TAIL-PCR, we found that the T-DNA in *nuwa/+* mutant was inserted into the single exon of the gene *At3g49240*, which contains a transposable element, *AT3TE74080* (Fig 2A). When the full-length genomic DNA of *At3g49240* was introduced into *nuwa/+*, homozygous mutant plants with normal fertility were obtained (Fig 2B), indicating that the phenotypes of *nuwa/+* were caused by loss of *At3g49240* function. *At3g49240* was designated as *NUWA*, the name of the Chinese goddess for human creation.

Sequence similarity and synteny analysis showed that *NUWA* is a single copy gene with high sequence similarity in different plant species (S2 Fig). We then investigated the nucleotide diversity of a 6-Kb region centered around *NUWA* in five Brassicaceae species, and found that the nucleotide diversity of the coding region was significantly lower than that of the promoter region and its 3’ downstream sequence; and also lower than genome-wide average (0.007) [37] (Fig 2C; S2 Fig). This relative low level of nucleotide diversity supports that NUWA is conserved, with its evolution driven by purifying selection.

We investigated *NUWA* expression patterns at both transcriptional and protein levels. First, real-time quantitative RT-PCR (qRT-PCR) analysis showed that *NUWA* was transcribed in all organs tested and was highly expressed in dry seeds and imbibed seeds (Fig 2D). We then generated *ProNUWA*:*NUWA*-GFP-GUS transgenic plants to test the protein distribution pattern of *NUWA*. In the inflorescence, GUS signals appeared at the tips of pistils and the petals of young flowers (Fig 2E). In ovules, GUS activity was detected on integuments before (Fig 2F) or early after fertilization (Fig 2G), in embryos at 2–4 cell stage (Fig 2G), heart stage (Fig 2H), torpedo stage (Fig 2I) and walking stick stage (Fig 2J) and later. In seedlings, GUS activity was detected in cotyledons, young leaves, root tips and lateral root primordia (Fig 2K and 2L). Little GUS activity was observed in mature leaves (Fig 2M). These results suggest that *NUWA* is highly expressed in the reproductive organs of *Arabidopsis*.

### *NUWA* is a maternal effect gene with its maternal allele specifically expressed before and after fertilization

**Fig 3:**
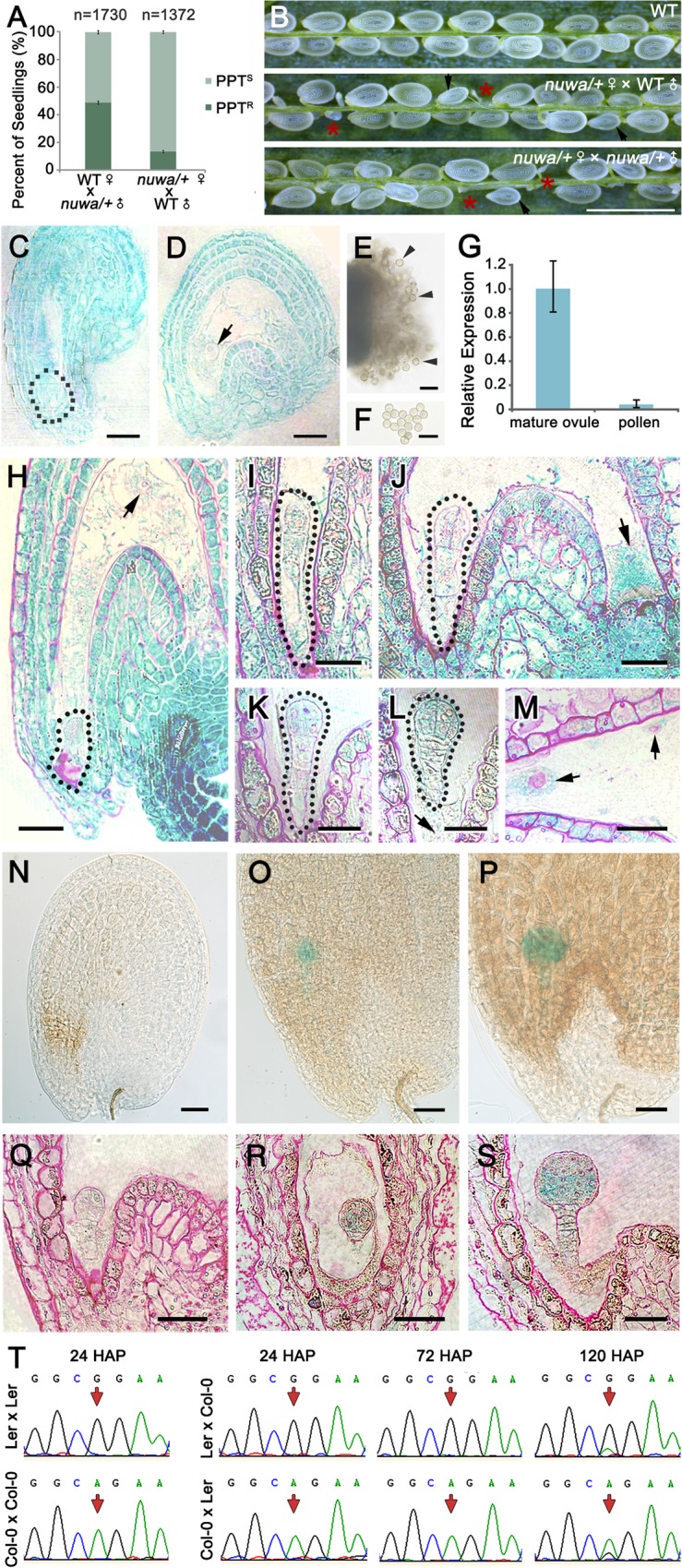
*NUWA* is a maternal effect gene that predominantly expresses its maternal allele before and after fertilization. (A) Reciprocal crosses between wild type and *nuwa/+*. Error bars, mean ± SEM. (B) Siliques of selfed wild type (upper), *nuwa/+* pollinated by wild type pollen (middle) and selfed *nuwa/+* (lower). Red stars and black arrows indicate early and late aborted seeds, respectively. Bar = 1 mm. (C-F) GUS staining in mature female (C-D) and male gametophytes (E-F) in *pNUWA*:*NUWA*-GFP-GUS transgenic plants. The dotted line and the arrow indicate the egg apparatus (C) and the central cell nucleus (D), respectively. Bars = 20 μm. No GUS activity was detected in pollen grains (E-F). Arrowheads indicate pollen grains. Bars = 50 μm. (G) Transcript levels of *NUWA* in mature ovules and mature pollen revealed by real-time qPCR. Error bars, mean ± SD. (H-M) GUS-stained ovules of wild-type pollen-pollinated homozygous *pNUWA*:*NUWA*-GFP-GUS transgenic plants at zygote stage (H), 1-cell embryo stage (I), 2/4-cell embryo stage (J), 8-cell embryo stage (K), 16-cell embryo stage (L and M). Basic fuchsin dye was used to mark the boundary of cells. The dotted lines and arrowheads mark the embryos and the endosperm or endosperm nuclei. Bars = 20 μm. (N-S) GUS-stained ovules of homozygous *pNUWA*:*NUWA*-GFP-GUS transgenic pollen-pollinated wild-type plants at the 8-cell (N and Q), the 16-cell (O and R) and the 64-cell stages (P and S). The ovules were observed by tissue clearing (N-P) or resin sections stained with basic fuchsin (Q-S). Bars = 30 μm. (T) Parental origin of *NUWA* transcripts in fertilized ovules at different developmental stages analyzed by allele-specific RT-PCR and Sanger sequencing. The polymorphism between Col-0 and Ler are indicated from self-crosses as a control in the far left column. The polymorphic nucleotide in each cross is indicated by a red arrow.

When reciprocal crosses were conducted between *nuwa*/+ and wild-type plants, male transmission was normal, while female transmission was severely reduced to 15.6% (Fig 3A). The decreased female gametophyte transmission rate, together with the observed embryo and endosperm defects, implies that *nuwa/+* is a maternal effect mutant. To verify this hypothesis, we pollinated *nuwa/+* with either wild-type or *nuwa/+* pollen. Seed-lethal phenotypes were observed in siliques (Fig 3B), and the percentage of aborted ovules were identical between the two crosses (|u| = 0.3255 < u_0.05_ = 1.96, *P* > 0.05) (S3A Fig), suggesting that the main phenotypes of the mutant seeds were controlled by the maternal genotype rather than that of both parents. These results confirmed that *nuwa/+* is a maternal effect mutant.

To elucidate the cause of the maternal effect in *nuwa/+*, we first investigated the expression of *NUWA* before fertilization. We transformed the *ProNUWA*:*NUWA*-GFP-GUS construct into *nuwa/+* to genetically complement the mutant and to investigate NUWA protein expression patterns in mature gametophytes in the wild-type background. GUS activity was observed in the egg apparatus (Fig 3C) and central cells (Fig 3D), but not in mature pollen located on the stigma (Fig 3E) or dispersed (Fig 3F). Real-time qPCR analysis showed that *NUWA* transcription levels were much lower in pollen than in mature ovules (Fig 3G). These results suggest that *NUWA* is predominantly expressed in female gametophytes before fertilization, with sparse expression occurring in male gametophytes.

We next analyzed the parent-of-origin-dependent expression patterns of the NUWA protein in ovules after fertilization. We reciprocally crossed homozygous *ProNUWA*:*NUWA*-GFP-GUS transgenic plants with wild-type ones. Maternal-specific GUS signal was detected in embryos and endosperm from the zygote to the 16-cell-embryo stage (Fig 3H to 3M). In contrast, a paternal-specific, embryo-specific GUS signal was not observed in early seeds (Fig 3N and 3Q) until the 16-cell-embryo stage (Fig 3O to 3P and 3R to 3S). These results indicate that only maternal-allele-specific expression of the NUWA protein is detected in early seeds.

To further analyze the parent-of-origin-dependent expression pattern of *NUWA* transcripts in ovules after fertilization, we adopted allele-specific RT-PCR. A single-nucleotide polymorphism (SNP) between *Arabidopsis* ecotypes Col-0 and L*er* in the exon of *NUWA* was used to distinguish parental origins of endogenous *NUWA* transcripts in seeds resulting from reciprocal crosses. We found that, for Col-0×L*er* cross, all the *NUWA* transcripts detected in 24 and 72 HAP were from Col-0 allele, and a small proportion of the transcripts detected in 120 HAP were from L*er* allele. Similarly, for L*er*×Col-0 cross, all the *NUWA* transcripts detected in 24 HAP and 72 were from L*er* allele, and a small proportion of the transcripts detected in 120 HAP were from Col-0 allele. These results indicate that, while transcripts derived from the maternal allele were detected in seeds from 24 HAP to 120 HAP, transcripts derived from the paternal allele were not detected in seeds until 120 HAP (Fig 3T). This result suggests that in the early seeds, only maternal-allele-specific *NUWA* transcripts are present in ovules, with transcripts of paternal allele first emerging between 72 HAP and 120 HAP. These data indicate that the maternal allele of *NUWA* is specifically detected at both transcriptional and translational levels before and shortly after fertilization.

### *NUWA* is an imprinted gene

**Fig 4:**
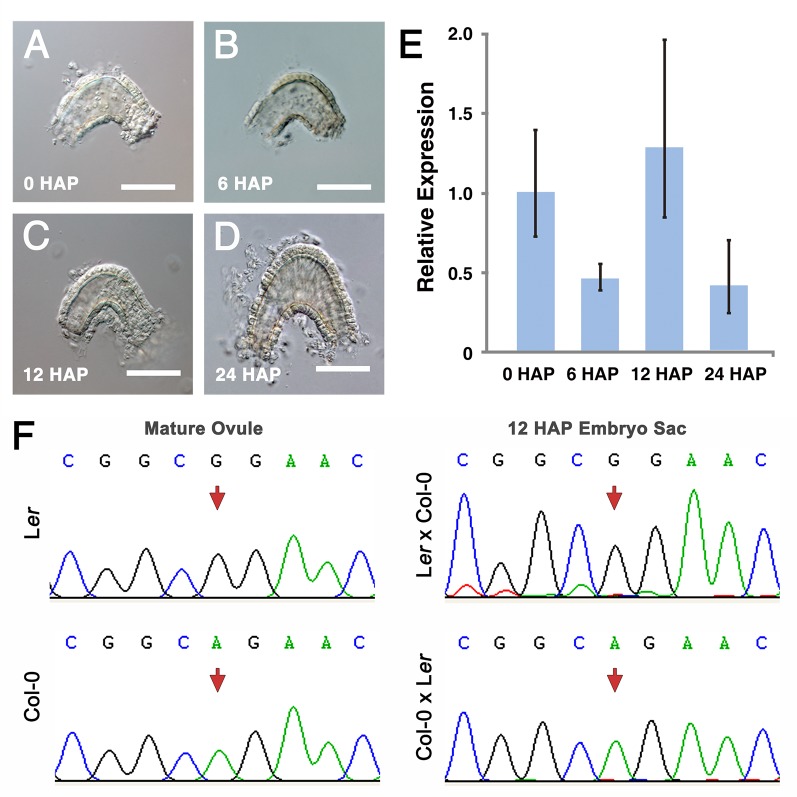
*NUWA* is an imprinted gene. (A-D) isolated embryo sacs at 0 HAP (A) (before fertilization), 6 HAP (B) (around fertilization), 12 HAP (C) and 24 HAP (D) (after fertilization). Bars = 50 μm. (E) Transcription level of *NUWA* in isolated embryo sacs by real-time qRT-PCR. Error bars, mean ± SD. (F) Parental origin of *NUWA* transcripts in isolated embryo sacs at 12 HAP analyzed by allele-specific RT-PCR and Sanger sequencing. The polymorphic nucleotides between Col-0 and L*er* are indicated by red arrows.

To precisely investigate the maternal expression pattern of *NUWA*, we analyzed our RNA-seq data of ovules at different developmental stages. We found that the amount of *NUWA* transcripts reduced at 6 HAP comparing to 0 HAP, then increased at 12 HAP, and decreased again at 24 HAP (S3B Fig). Because fertilization is completed within 6 HAP in *Arabidopsis* [38], this result reveals that *NUWA* is transcribed in ovules after fertilization. As *NUWA* expresses on integuments at early development stages, we then isolated wild type embryo sacs at 0 HAP, 6 HAP, 12 HAP and 24 HAP (Fig 4A to 4D) and examined transcripts of *NUWA*. The qRT-PCR results showed the transcript level of *NUWA* in embryo sacs decreased at 6 HAP before increasing at 12 HAP (Fig 4E), which confirmed that *NUWA* is transcribed *de novo* in embryo sacs after fertilization. Because the paternal allele of *NUWA* transcript is not detected before 120 HAP (Fig 3T), this result suggests that the *de novo* expressed *NUWA* transcripts are predominantly from the maternal allele. Since imprinted genes are known as genes expressed predominantly from one of their parental alleles during a period of diploid stage [2], our results also suggests that *NUWA* is an imprinted gene. To confirm this hypothesis, we isolated the embryo sacs at 12 HAP from progeny plants of reciprocal crosses between wild type Col-0 and L*er*, extracted RNA and performed allele-specific RT-PCR. The results showed that in 12 HAP embryo sacs, only maternal-allele-specific *NUWA* transcripts were detected (Fig 4F), which supports that the *de novo* expression of *NUWA* transcripts detected in early developmental stage are predominantly from the maternal allele. These results indicate that *NUWA* is a maternally expressed imprinted gene.

### NUWA is localized to mitochondria and is required for mitochondrial development and function

**Fig 5:**
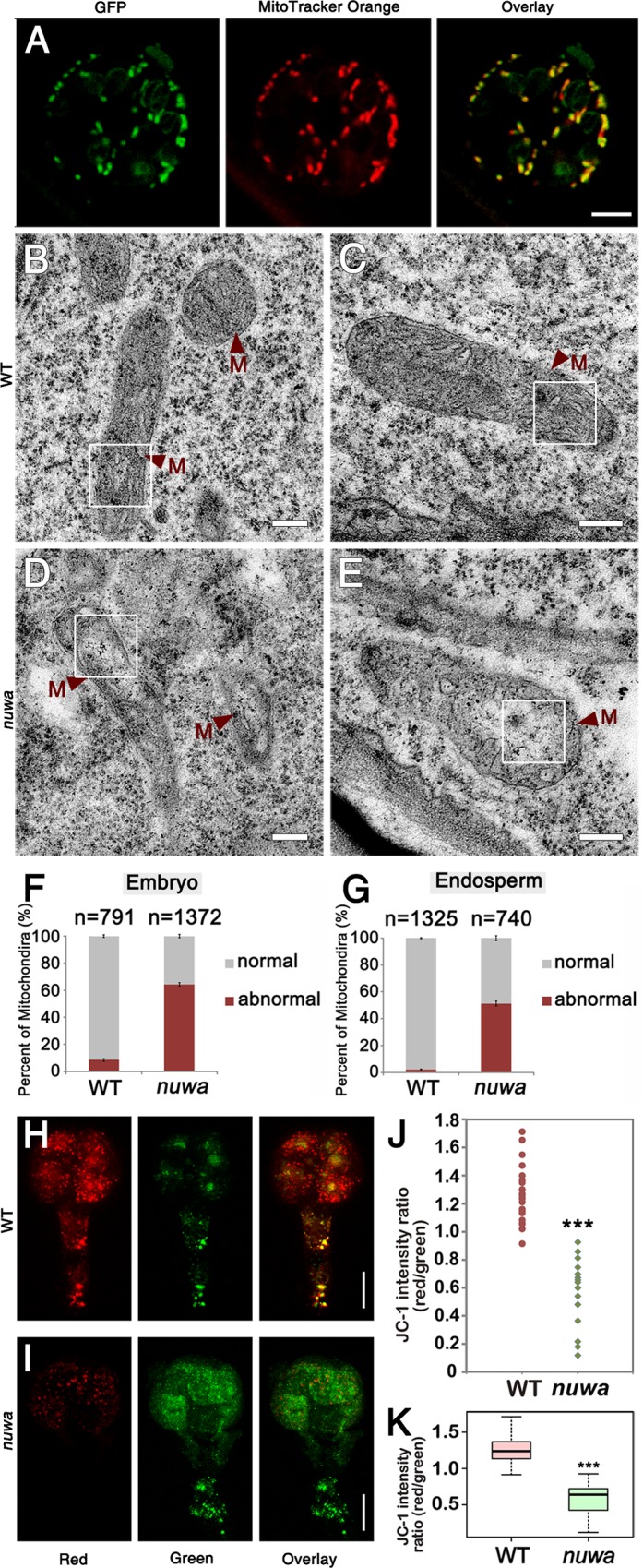
NUWA is a mitochondrial-localized protein that is required for mitochondrial development in the early embryo and endosperm. (A) GFP signals, MitoTracker Orange signals and the overlay of both signals in a guard cell of *pNUWA*:*NUWA*-GFP-GUS transgenic seedling. Bar = 5 μm. (B-C) Mitochondria in embryos (B) and endosperm (C) in ovules of wild-type plant. M, mitochondria. Bars = 200 nm. (D-E) Mitochondria in embryos (D) and endosperm (E) in aborted ovules of *nuwa/+* plant. M, mitochondria. Bars = 200 nm. (F-G) Percentage of normal and abnormal mitochondria in embryos (F) and endosperm (G) in wild-type and aborted *nuwa/+* ovules. Error bars, mean ± SEM. (H-I) JC-1 fluorescence in isolated wild type embryo (H) and *nuwa/+* mutant embryo (I) at the 8-cell stage. Bars = 10 μm. (J-K) Dispersion graph (J) and box-plot (K) showing the red/green JC-1 fluorescence ratios measured in isolated wild type embryos (n = 22) and *nuwa/+* embryos (n = 15) at various developmental stages (from the 2-cell stage to the 16-cell stage). Asterisks indicate statistically significant differences compared with the wild type. (***, P<0.01). White frames are the regions been enlarged in S6 Fig.

When we investigated the subcellular localization of NUWA, the GFP fluorescence signal was found to co-localize with the mitochondria stained with the specific dye MitoTracker Orange in seedling cells (Fig 5A). The similar GFP signal was also observed in the heart-stage embryo, and the signal did not co-localize with the plastid auto-fluorescence (S4 Fig). Additionally, a putative mitochondrial-targeting peptide (mTP) was predicted in the N-terminus of NUWA [39]. These results suggest that NUWA is localized in mitochondria. When we introduced a *ProNUWA*:*NUWA*ΔmTP-GFP into *nuwa/+*, the mitochondrial-co-localized GFP signal disappeared although the GFP transcript was detected (S5 Fig), implying that mitochondrial localization of NUWA is mediated by the mTP. In addition, no homozygous *nuwa* was identified among 180 T_2_ transformants, demonstrating that the NUWAΔmTP-GFP fragment could not rescue *nuwa*. These results suggest that the mitochondrial localization of NUWA is essential for its proper function.

To investigate whether *NUWA* affects mitochondrial development, we adopted transmission electron microscopy (TEM) to analyze ultrastructure in early embryos and endosperm. Compared with mitochondria in wild-type embryos (Fig 5B) and endosperm (Fig 5C), the mitochondrial matrix of *nuwa* mutant embryos and endosperm was less dense, with the cristae composed of structurally looser inner membranes (Fig 5D and 5E, S6 Fig). Statistical analysis showed that the percentages of abnormal mitochondria in *nuwa/+* mutant embryos and endosperm were 64.3% and 51.4%, respectively, both significantly higher than those in wild-type plants (8.60% and 2.19%, respectively) (Fig 5F and 5G) (|u| = 25.15 > u_0.01_ = 2.58, *P* <0.01; |u| = 26.88 > u_0.01_ = 2.58, *P* < 0.01). This result indicates that abnormal mitochondria were significantly more abundant in *nuwa* mutant ovules.

We then investigated mitochondrial functional status in isolated early embryos by using the mitochondrial trans-membrane potential indicator JC-1. In healthy cells with mitochondria at high membrane potential, JC-1 exhibits red fluorescence; in abnormal cells with mitochondria at low membrane potential, JC-1 exhibits green fluorescence [40,41]. Results show that red signals of JC-1 were visibly stronger in many wild type embryos (Fig 5H and S7 Fig), whereas JC-1 exhibited much stronger green signals in many *nuwa* mutant embryos at the same development stages (Fig 5I and S7 Fig). Quantitative analysis showed that the red-to-green fluorescence ratios of *nuwa* embryos were significantly lower than those of wild type embryos (|t| = 9.277 > t_0.005_ = 3.591, P < 0.005) (Fig 5J and 5K), indicating that the mitochondrial membrane potential levels of the mutant embryos were decreased compared to the wild type embryos. This result suggests that the mutant embryos of *nuwa*/+ have defective mitochondria, and the abnormal phenotypes of mutant embryos and endosperm might be attributed to mitochondrial dysfunction.

Because PPR proteins are widely involved in regulating the post-transcriptional processing of organelle encoded genes through affecting splicing, editing and translation of organelle transcripts [42,43], we intend to investigate whether NUWA is involved in regulating the expression level and splicing of mitochondrial genes. We extracted RNA from isolated endosperm of *nuwa* mutant and wild type plant at the same developmental stage, and analyzed, by using real-time qPCR, the expression level of those intron-containing mitochondrial genes and of those ovule-highly expressed genes chosen based on our preliminary RNA-seq data and the expression pattern data in Arabidopsis eFP Browser Database [44]. We also analyzed the trans splicing of the three NAD1 genes, two NAD2 genes and three NAD5 genes. The result showed that the expression levels of these genes were higher in the *nuwa* mutant, and no defect in splicing was detected (S8 Fig), suggesting that the expression level of mitochondria encoded genome is abnormal in the mutant, and that NUWA is probably not involved in splicing and stabilization processes of mRNA encoded by mitochondria.

### The first PPR motif and the coiled-coil domain are essential to the function of NUWA during embryogenesis and endosperm development

**Fig 6:**
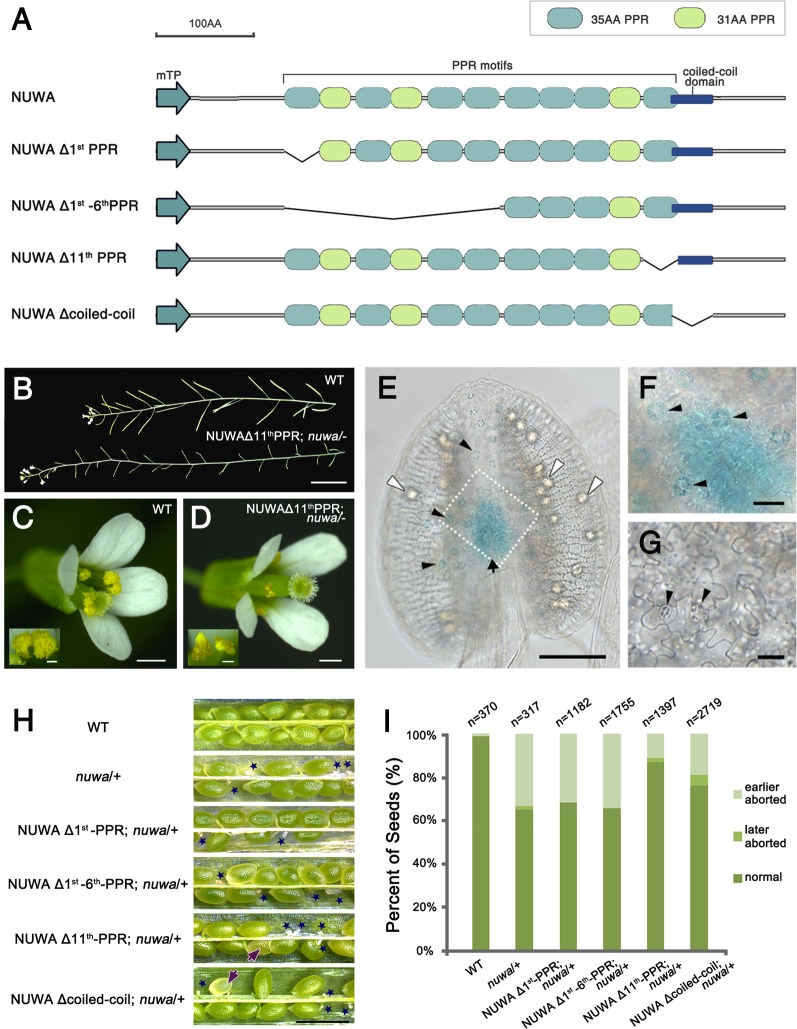
The 1^st^ PPR Motif and the Coiled-Coil Domain Are Critical for the Proper Function of NUWA. (A) The deletion variants of NUWA. (B) The wild type and *nuwa*/- plants complemented by NUWAΔ11^th^PPR. Bar = 2 cm. (C-D) Flowers of wild-type (C) and *nuwa*/- plants complemented by NUWAΔ11^th^PPR (D). Bars = 500 μm. The small pictures at the corners are enlarged images of anthers. Bar = 100 μm. (E) GUS signal of *pNUWA*::*NUWA*-GFP-GUS was detected in the joint region of anther and filament, and the guard cells on the anther wall. Black arrow points to the joint region of anther and filament, black arrowheads point to the guard cells, white arrowhead points to the pollen. Bar = 100 μm. (F) Enlarged view of the region surrounded by the white dotted line in (E). Black arrowheads point to the guard cells. Bar = 20 μm. (G) GUS signal of *pNUWA*::*NUWA*-GFP-GUS was not observed in guard cells on mature leafs. Black arrowheads point to the guard cells. Bar = 20 μm. (H) Seed sets of wild type, *nuwa*/+ and *nuwa*/+ plants transformed with four deletion variants. Blue stars and purple arrows indicate earlier and much later aborted seeds, respectively. Bar = 1 mm. (I) Seed abortion rate of wild type, *nuwa*/+ and *nuwa*/+ plants transformed with four deletion variants.

*NUWA* encodes a P-subfamily pentatricopeptide repeat (PPR) protein of 629 amino acids with 11 PPR motifs (Fig 6A). PPR proteins, which form one of the largest protein families in higher plants, are named after the eukaryote-specific PPR repeat that typically comprises 35 amino acid residues, and have RNA-binding activities [45,46]. Not all PPR motifs contribute directly to the RNA-binding activity of PPR proteins, and some PPR proteins have protein-binding activity [46–48]. We therefore investigated whether all the PPR motifs in NUWA are equally important to its function. We also examined the importance of the predicted coiled-coil domain, which usually functions in protein-protein binding. We created four GUS-tagged deletion variants of NUWA, each expressed with the *NUWA* promoter, for genetic complementation (Fig 6A). We observed that NUWAΔ1^st^PPR, NUWAΔ1^st^-6^th^PPR and NUWAΔcoiled-coil failed to genetically complement *nuwa*/+ (S1 Table). No homozygous *nuwa* mutant was identified out of 200 or 180 T_2_ resistance-pre-selected transformants of each variant; in contrast, a NUWA-GUS fusion protein generated in the same manner completely rescued *nuwa*. These results indicate that both the first PPR motif and the coiled-coil domain are crucial to NUWA protein functions during early embryogenesis and endosperm development.

When we used NUWAΔ11^th^PPR to complement *nuwa*/+, however, 10 homozygous *nuwa* were detected out of 200 T2 resistance-pre-selected transformants (S1 Table). These homozygous *nuwa* that were complemented by NUWAΔ11^th^PPR-GUS had longer stems, undeveloped siliques (Fig 6B) and flowers featuring shortened stamens and shriveled anthers with undeveloped pollen (Fig 6C and 6D). These results indicate that the 11^th^ PPR motif is not critical to NUWA function during embryogenesis and endosperm development. However, NUWA may have functions beyond embryogenesis and endosperm development, as the GUS signals were detected in the junction region of filament anthers (Fig 6E) and specifically in stomatal guard cells on anther walls (Fig 6E to 6G) of *proNUWA*::*NUWA*-GFP-GUS transgenic plants.

We next examined seed-setting rates and arrested seed developmental stages in the T2 transgenic plants in the *nuwa*/+ background. Seeds aborted as early as those of *nuwa*/+ were observed in siliques of these transgenic plants, whereas later-aborted seeds were found in siliques of transgenic plants expressing NUWAΔ11^th^PPR or NUWAΔcoiled-coil fragments (Fig 6H). Deeper analysis revealed that rates of seed abortion in heterozygous transgenic plants expressing NUWAΔ11^th^PPR and NUWAΔcoiled-coil were 13.0% and 23.9%, respectively, which is significantly lower than the abortion rate of *nuwa*/+ in this experiment (Fig 6I). This result confirms that the 11^th^ PPR motif of NUWA is not as important as the other motifs and domains that were analyzed for their biological functions in the protein. These data also indicate that the coiled-coil domain is functionally less crucial than the first PPR motif during embryogenesis and endosperm development.

## Discussion

In this study, we have identified the expression pattern and essential role of a novel imprinted gene, *NUWA*, in *Arabidopsis* early seed development. First, *NUWA* is an essential gene that controls early embryogenesis and endosperm development in *Arabidopsis*, because loss of function of *NUWA* resulted in embryo and endosperm lethality. Second, *NUWA* is a maternal effect gene, because control of seed development is only through the maternal allele of *NUWA*, and wild-type pollen could not rescue the defective phenotypes. Third, *NUWA* is imprinted, as only the maternal allele-specific *NUWA* transcripts and proteins were identified before and early after fertilization, and the maternal allele of *NUWA* is transcribed *de novo* after fertilization. Fourth, *NUWA* is a new type of imprinted gene, because NUWA is localized in the mitochondria and is essential for the proper function of the mitochondria.

Only the maternal allele-specific *NUWA* transcripts and encoded proteins were detectable during a period of several days after fertilization, and *de novo* transcription of *NUWA* started shortly after fertilization. As imprinted genes feature parent-of-origin specific allele biased expression patterns [2,7], or, in particular, parental allele-specific *de novo* transcription after fertilization [16], we concluded that *NUWA* is an maternally expressed imprinted gene. In addition, a TE is inserted in the coding sequence of *NUWA*, and most of the identified imprinted genes in *Arabidopsis* have TEs flanked by or harbored in coding sequences [29,49]. The mechanism of how *NUWA* imprinting is regulated, however, is not yet clear, and needs to be further investigated.

*NUWA* is a new type of imprinted gene. It is not because that *NUWA* is not only expressed in the endosperm but also expressed in many other tissues (Fig 3H to 3M). In fact, although some of the plant imprinted genes are only expressed in endosperm (such as *FWA*), a lot of plant imprinted genes are expressed in multiple tissues. For instance, *MEADA*, one of the most famous imprinted genes in plant, was expressed in embryo and in many other vegetative tissues from both paternal and maternal allele [20,50]. In addition, genes expressed and imprinted in embryo were also discovered in maize [51], rice [32] and *Arabidopsis* [16]. *NUWA* is special because its expression starts before fertilization, and the maternal control of it in early seed development is mediated by both maternally-deposited products and genomic imprinting. Maternal-specific *NUWA* products (including both RNA and protein) are detected in mature gametes before pollination, suggesting that maternal-deposited products of *NUWA* exist in embryo sac shortly after fertilization. Because *NUWA* encodes a PPR protein and mRNAs of PPR protein-encoding genes were reported to be rather unstable with a much shorter half-life (*i*.*e*., less than 6 hours) than the average mRNA half-lives of other genes [52], it is possible that the maternal allele of the zygotic *NUWA* is specifically activated soon after fertilization to make up the shortage of maternally-deposited *NUWA* products degraded during the maternal-to-zygotic transition [53–55]. Therefore, the products of *NUWA* detected in embryo sac early after fertilization are composed of the maternally-deposited *NUWA* products expressed in female gametophyte before fertilization and the maternal-allele specific *NUWA* products *de novo* synthesized after fertilization. That is, the parental effect of *NUWA* is controlled by parental-allele-specific expression not only before but also after fertilization, which is different from other reported imprinted genes in plants.

It is not yet known whether the imprinting of *NUWA* occurs in embryo, or in endosperm or in both. The imprinting of *NUWA* occurs very early, *i*.*e*., at about zygote stage, and *NUWA* is expressed in integument and seed coat. Unfortunately isolation and collection of enough amounts of *Arabidopsis* zygote and endosperm at zygote stage are extremely challenging, making it difficult to investigate the region in *NUWA* where imprinting occurs and the imprinting mechanism. Even so, we still think that *NUWA* is very likely to be imprinted in embryo. The expression of maternal allele of *NUWA* (*i*.*e*., the GUS signal) could always be detected in embryos staged from zygote stage to later stages, whereas the expression of paternal allele of *NUWA* could not be detected in embryo until the 16-cell stage, suggesting that the *NUWA* products in early embryo are predominantly derived from its maternal allele. From the TEM result, we could observe severe defective phenotypes in mitochondria in *nuwa* embryos, suggesting an important function of *NUWA* in mitochondria and in early embryogenesis. Meanwhile, NUWA belongs to PPR family, proteins from which all have very short half-lives [52]. The *de novo* expression of the maternal allele of *NUWA* is likely to be present in the embryo to make up the shortage of maternally-deposited *NUWA* products degraded during the maternal-to-zygotic transition. Therefore it is very possible that *NUWA* is imprinted in embryo. On the other hand, *NUWA* is also very possible to be imprinted in endosperm. The products of the paternal allele of *NUWA* were never detected in endosperm. It is likely that the products of *NUWA* in early endosperm are also predominantly derived from its maternal allele. Similarly, from the TEM results we could also see that *NUWA* has important function in early endosperm development. As NUWA protein could be detected in endosperm from zygote stage to 16-cell stage, the *de novo* expression of the maternal allele of *NUWA* is also likely to be present in the endosperm. Thus *NUWA* is also likely to be imprinted in endosperm.

*NUWA* is a new type of imprinted gene, also because it functions in mitochondria. In plants, although many imprinted genes have been identified, functional imprinted genes are still rarely discovered [17]. *NUWA* is a functional imprinted gene identified in *Arabidopsis* which controls early embryo and endosperm development. In addition, *NUWA* is the first discovered imprinted gene in plants whose protein product has an essential function in mitochondria. In mammals, imprinted genes that function in mitochondria were also identified, loss of function of which lead to severe diseases [56–58]. The discovery of *NUWA* indicates that, like the imprinted genes in mammals, imprinted genes in plants are also involved in many aspects of subcellular biological processes during early embryo development.

The specific mitochondria RNAs that are regulated by NUWA remain to be characterized. As we did not find reduced expression level and defected splicing in mitochondrial mRNA of the mutant (S8 Fig), it is possible that *NUWA* might be involved in regulating translation of mitochondrial mRNA, possibly through binding to mitochondrial mRNA and facilitating the release of a hairpin structure without changing RNA sequence and RNA copy number [59,60]. Another possibility is that *NUWA* may participate in the post-transcriptional processing of tRNAs or rRNAs. However, it is extremely challenging to detect the structural change of mitochondrial mRNAs, and the sequence and expression level changes of mitochondrial tRNAs and rRNAs, especially from the materials of early embryo, endosperm or ovules of *Arabidopsis*. New technology advances would be required to investigate the specific molecular function of *NUWA* in mitochondria.

The mitochondrion is an important organelle for energy and metabolism in eukaryotic cells. Coordination between mitochondrial and nuclear genomes is important for the efficiency of mitochondrial function, because mitochondrial proteins encoded by both genomic and mitochondrial DNA varies significantly in different cells [61–63]. In most species of higher eukaryotes, mitochondria are maternally inherited after fertilization. In *Arabidopsis*, mitochondrial DNA in sperm cells is almost completely degraded before fertilization [63]. Moreover, very few mitochondria from *Arabidopsis* sperm cells are able to enter the embryo sac during double fertilization, and all are degraded shortly after gamete fusion, as in animals [64,65]. Therefore, all the mitochondria and mitochondrial DNA in the fertilized *Arabidopsis* embryo sac are maternally inherited. After fertilization, a series of remarkable changes take place in newly formed zygotes and endosperm, including recombination of the two differentiated nuclear genomes, remodeling of parental chromatin, and fusion and coordination of parental nuclear and cytoplasmic components [54,55,66]. Consequently, regulating maternally inherited essential mitochondria by nuclear-encoded maternal allele-specific *NUWA* products could possibly improve the implementation efficacy of the *NUWA* function and increase the efficiency of mitochondria-nuclear interaction in the complex post-fertilization environment, which might be the advantage for having an essential gene *NUWA* to be imprinted and selected during evolution. Therefore, the imprinting of *NUWA* would be an adaptation and enhancement to the maternal control of mitochondrial development. The discovery of *NUWA* reveals a unique mechanism of maternal control of the mitochondria and adds an extra layer of complexity to the regulation of nucleus-organelle coordination during early plant development.

## Methods

### Plant materials and growth conditions

*Arabidopsis thaliana* ecotype Columbia-0 (Col-0) was used as wild type plant. All the transgenic lines used in this study were in the Columbia ecotype. Wild type *Arabidopsis thaliana* Landsberg *ereta* (L*er*-0) was only used in the allele specific RT-PCR. The *nuwa* mutant was obtained from the T-DNA insertion mutant library (in Col background) of our lab [34]. Plants were grown under long-day conditions (16 hr light/8 hr dark) at 22°C. For crosses, maternal partners were emasculated, and pollinated 2 or 3 days after emasculation.

### TAIL-PCR and genotype analysis

The flanking sequence of the T-DNA insertion site in *nuwa* was determined by TAIL-PCR with the specific and arbitrary degenerate primers described previously [34]. For genotypic analysis of *nuwa*, primers *nuwa*_CS_F and *nuwa*_CS_R were used to amplify the wild-type allele of *NUWA*. Primers *nuwa*_CS_R and DL3 were used to amplify the insertion allele of *nuwa* and described in the S2 Table.

### RNA extraction and qPCR analysis

Total RNA of most tissues was extracted from liquid nitrogen frozen tissues using TRIzol (Invitrogen) according to manufacturer’s instructions. Total RNA extraction of seeds and stem was performed with phenol extraction buffer (50% Tris saturated phenol, 0.5% SDS, 50 mM LiCl, 50 mM Tris-HCl at pH8.0, 50 mM EDTA at pH8.0) at room temperature. Residual DNA was removed using the RNase-free DNase (TaKaRa), and cDNA was synthesized using the M-MLV kit (Invitrogen/Fermentas) according to the manufacturer’s instructions.

In real-time qPCR, diluted cDNA was used as a template, and three biological repeats was performed using SYBR Green real-time PCR Master Mix (TOYOBO) as described previously [67] on an ABI 7500 Real-Time PCR System. The relative expression level of each gene was calculated with the cycle threshold (CT) 2^-ΔΔCT^ method [68]. Gene expression values were standardized to TUB2 (AT5G62690) or *PP2AA3* (AT1G13320).

### Microscopy analysis

DIC observation of cleared ovules and bright field observation of GUS stained ovules were performed using Olympus BX51 microscope. Images of other GUS stained samples were taken using a Leica M205C FA stereoscope.

Subcellular localization observation was performed using a confocal laser scanning microscope (Leica TCS SPE confocal microscope).

To determine the phenotype of endosperm nuclei, confocal observation of ovules was modified from previously described [69]. Artificial pollinated selfed 26 HAP siliques were harvested and fixed in 4% glutaraldehyde (in 12.5 mM cacodylate, pH 6.9), and a vacuum was applied for the initial 20 min, after which they were in fixative overnight at room temperature. After fixation, samples were dehydrated through a conventional ethanol series with 20 min per step, then cleared in 2:1 (v/v) benzyl benzoate:benzyl alcohol for a minimum of 1 hr. Siliques were dissected, mounted with immersion oil, and observed with a Leica TCS SPE confocal microscope.

For transmission electron microscopy, protocol for sample preparation was modified from previously described [70]. Developing seeds at specific stages were collected, vacuumed and fixed in 5% glutaraldehyde and 3% paraformaldehyde in 0.1 M sodium cacodylatetrihydrate buffer (pH7.2) at room temperature overnight and then at 4°C for 24 hrs. Ovules were then washed four times with 0.1 M sodium cacodylatetrihydrate buffer (more than 30 min for each time) and fixed in 2% O_S_O_4_ at room temperature for 4 hrs and then at 4°C over night, followed by dehydration in a graded ethanol series. Seeds were embedded in a complete resin mixture (Spi-chem Spurr) and incubated in 65°C for 16 hrs. Samples were sectioned using a Leica EM UC6 ultramicrotome and stained with 2% uranyl acetate and lead stain solutions. Sections were examined with a FEI Tecnai G^2^ 20 Twin electron microscope at 200 kV.

### Plasmids construction and plant transformation

For genetic complementation, we used GW_*NUWA*_COMP_16_F and GW_*NUWA*_COMP_R primers to amplify the *NUWA* genomic DNA and generated the *pNUWA*::*NUWA* genomic DNA constructs through BP clonase and subsequent LR clonase (Invitrogen). The amplified fragments were cloned into a reconstructed vector pK7FWG0, which was made from the GATEWAY-compatible destination vector pB7RWG2 (Department of Plant Systems Biology, VIB-Ghent University, Ghent, Belgium) by digesting the 35S promoter *via Spe* I and *Sac* I sites.

To generate the *pNUWA*::*NUWA*-EGFP-GUS constructs, the *NUWA* genomic DNA was amplified using TOPO_*NUWA*_GUS_F and TOPO_*NUWA*_pg+GUS_R primers. This genomic sequence was cloned into pBGWFS7 vector (Department of Plant Systems Biology, VIB-Ghent University, Ghent, Belgium) through pENTR/D-TOPO kit (Invitrogen) and LR reactions.

In the genetic complementation assay with truncated NUWA, five groups of sequences were amplified to generate the *pNUWA*::*NUWA*ΔmTP, *pNUWA*::*NUWA* Δ1^st^-PPR, *pNUWA*::*NUWA* Δ1^st^to6^th^-PPR, *pNUWA*::*NUWA* Δ11^th^-PPR and *pNUWA*::*NUWA* Δcoil constructs. As NUWA has no intron, genomic DNA was used as templates in the amplifications. P4P1R_NUWAΔX_F and P4P1R_NUWAΔX_R (X represents one of mTP, 1^st^-PPR, 1^st^to6^th^-PPR, 11^th^-PPR and coil) primers were used to amplify the sequence of the promoter. This sequence was then cloned into pDONRP4-P1r by BP reaction to generate pEN-L4-NUWAΔX-R1. Meanwhile, TOPO_NUWAΔX_F and TOPO_NUWAΔX_R primers were used to amplify the sequence from the base next to the coding region of the mTP to the base right before the terminator. This sequence was then cloned into pENTR/D-TOPO by TOPO reaction to generate pENTR-NUWAΔX. GUS and EGFP coding sequence were amplified by GUS-F_attB2r/ GUS-R_attB3 and EGFP-F_attB2r/ EGFP-R_attB3 primers, and cloned in to pDONRP2R-P3 respectively to generate pEN-R2-GUS-L3 and pEN-R2-EGFP-L3. The *pNUWA*::*NUWA*ΔX constructs were generated by LR reaction of four plasmids, including pK7m34GW, pEN-L4-NUWAΔX-R1, pENTR-NUWAΔX, and pEN-R2-EGFP-L3.

Constructs were transformed into *Agrobacterium tumefaciens* GV3101, using a freeze-thaw procedure. *Arabidopsis* transformation and transgenic plant screening were conducted as reported [71].

### Staining and tissue clearing

The histochemical GUS staining was modified from described previously [72]. After per-fixation by pure acetone at -20°C for 1 hr, tissues immerged in a staining solution containing 0.5 mg ml^-1^ X-gluc (Sigma) in 100 mM Na_2_HPO_4_/NaH_2_PO_4_ (pH7), 2 mM K_3_Fe(CN)_6_, 2 mM K_4_Fe(CN)_6_, 10 mM EDTA and 0.1% Triton X-100, modified from [35]. Samples were vacuumed for 10 min and then put in a 37°C incubator overnight. The staining buffer was removed, and the samples were fixed in FAA for 1 or 2 hrs before cleared and embedded.

For fuchsin basic staining, resin sections on the glass slides was covered by drops of 1% fuchsin basic solution and incubated at 60°C for 6 min. Then wash away the dye by flowing sterile water. Slices were sealed with 50% glycerine before microscopy.

MitoTracker Orange (Invitrogen) staining of the seedlings was performed according to manufacturer’s instructions, as described before [73].

The GUS stained samples were cleared using 70% ethanol before microscopy.

For whole-mount preparations, ovules at different development stages were mounted in diluted Hoyer’s solution, which was introduced on http://www.seedgenes.org/Tutorial.html [74], and cleared for 5 to 30 min before microscopy analysis.

### Mitochondrial membrane potential analysis

Isolated early embryos were incubated in 12% (w/v) mannitol with 10 μg/mL JC-1 (Molecular Probes, Invitrogen) for 30 min at room temperature, and then washed with 12% (w/v) mannitol. Images were collected using the confocal microscope mentioned above. JC-1 aggregates were detected with red fluorescence (excitation, 532 nm; emission, 575–590 nm), and JC-1 monomers were detected with green fluorescence (excitation, 488 nm; emission, 515–545). The ratio of red to green fluorescence of JC-1 images was calculated by Imaris 7.5.0 software (Bitplane). R software was used for the box-plot.

### Allele specific RT-PCR

cDNA from ovules (or isolated embryo sacs) at different development stages from self and crossed Col-0 and L*er* was used for allele-specific RT-PCR (primers and PCR parameters in S2 Table). PCR products were sequenced by the Sanger Chain Termination Method.

### Embryo sac isolation, RNA extraction and cDNA synthesis of isolated embryo sacs

The isolation of embryo sacs at different stages and the extraction of RNA from the isolated embryo sacs were performed according to modified versions of protocols described previously [75]. Embryo sacs were isolated by brief manual dissection combined with enzymatic maceration. Enzymatic solution is composed of 1% cellulose (Yakult Honsha Co. Ltd, Tokyo, Japan) and 0.8% Macrozyme R-10 (Yakult Honsha). Before manual dissection, ovules at different development stages were incubated in the enzyme solution for 0.5 h. The Dynabeads mRNA DIRECT Micro Kit (Invitrogen) was used for mRNA isolation. cDNA was synthesized using the SuperScript III kit (Invitrogen) according to the manufacturer’s instructions. For each experiment (or each time point), three independent biological replicates were performed. For each independent biological replicate, 11–16 embryo sacs were isolated for RNA extraction.

## Supporting Information

S1 Fig Female gametophytes could be fertilized in *nuwa/+* mutant. (A and B) Ovules with GUS signal resulting from pollination of emasculated wild type (A) and *nuwa-1/*+ (B) by pollen of FAC1-GUS marker line. Bars = 100 μm. (C) Statistic analysis of ovules with GUS signal resulting from pollination of emasculated wild type and *nuwa-1/+* by pollen of FAC1-GUS marker line. (D-I) FIS2-GUS marker line in wild type background. GUS signals indicate the endosperm nuclei. Bars = 50 μm.
(TIF)

S2 Fig *NUWA* is a conserved gene. (A) Synteny analysis of *NUWA* in plant species. Colors of the arrows represent for the various genes in the flanking regions of *NUWA*. Syntenic orthologs are depicted in the same color, while white arrows are functionally uncharacterized genes. The grey arrows aligned in the middle are *NUWA* gene and its orthologs in the same direction in each genome. Topology shown here is a species tree based on Phytozome v10. (B) Phylogenetic tree of *NUWA* and its orthologs. Scale bar, 0.2 amino acid substitutions per site. Ath, *Arabidopsis thaliana*; Aly, *Arabidopsis lyrata*; Cru, *Capsella rubella*; Bra, *Brassica rapa*; Esa, *Eutrema salsugineum*; Gra, *Gossypium raimondii*; Tca, *Theobroma cacao*; Ccl, *Citrus clementina*; Egr, *Eucalyptus grandis*. (C) Nucleotide diversity in the genomic region centered on *NUWA* among 19 *A*. *thaliana* accessions: Col-0, Bur-0, Can-0, Ct-1, Edi-0, Hi-0, Kn-0, Ler-0, Mt-0, No-0, Oy-0, Po-0, Rsch-4, Sf-2, Tsu-0, Wil-2, Ws-0, Wu-0 and Zu-0. (D) Tajima’s *D* value along the 6 kb genomic region centered on *NUWA* based on the same set of 19 *A*. *thaliana* accessions.
(TIF)

S3 Fig *NUWA* is a maternally expressed imprinted gene. (A) Percentage of seeds in wild type and *nuwa-1/+* self crossed siliques and siliques resulted from emasculated *nuwa-1/+* pollinated by wild type pollen. (B) RNA-seq data of the transcription level of *NUWA* in ovules at different developmental stages.
(TIF)

S4 Fig NUWA is not localized in plastids. (A-D) GFP signals (A), autofluorescence of chloroplast (B), the overlay of both signals (C) and bright field (D) in heart-stage embryo of *pNUWA*:*NUWA*-GFP transgenic plant. Bar = 20 μm.
(TIF)

S5 Fig NUWA is localized in mitochondria by its mitochondrial target peptide. (A-C) GFP signals (A), MitoTracker Orange signals (B) and the overlay of both signals (C) in root cells of *pNUWA*:*NUWA*-GFP transgenic seedling. Bar = 5 μm. (D-F) GFP signals (D), MitoTracker Orange signals (E) and the overlay of both signals (F) in root cells of *pNUWA*:*NUWA*ΔmTP-GFP transgenic seedling. Bar = 5 μm. (G) The expression level of GFP in seedling of five *pNUWA*:*NUWA*ΔmTP-GFP transgenic lines and one of the *pNUWA*::*NUWA*-GFP-GUS transgenic lines. The genomic GFP sequence and the *tubulin2* sequences were amplified as controls.
(TIF)

S6 Fig Enlarged parts of mitochondria in Fig 5 showing the density of mitochondrial matrix and the cristae composed of inner membranes. (A) Enlarged part of mitochondria in Fig 5B marked by the white frame. (B) Enlarged part of mitochondria in Fig 5C marked by the white frame. (C) Enlarged part of mitochondria in Fig 5D marked by the white frame. (D) Enlarged part of mitochondria in Fig 5E marked by the white frame. White arrowheads indicate the inner membranes.
(TIF)

S7 Fig Mitochondrial functional status in isolated early embryos indicating by the mitochondrial membrane potential indicator JC-1. (A-B) JC-1 fluorescence in isolated wild type embryos (A) and *nuwa-1/+* mutant embryos (B) at different developmental stages. For each embryo, green fluorescence, red fluorescence, overlay the two fluorescence and bright field are shown. Bars = 10 μm.
(TIF)

S8 Fig Splicing and expression pattern of mitochondrial genes which have introns or highly expressed in ovules revealed by real-time qPCR. Error bars, mean ± SD.
(TIF)

S1 Table Genetic complementation of *nuwa*/+ by four deletion variants of NUWA. (XLSX)

S2 Table Primers used in this study. (XLSX)
