# Supplementary figures and images for: A Novel Imprinted Gene NUWA Controls Mitochondrial Function in Early Seed Development in Arabidopsis
Source: PLoS Genet. 2017 Jan 17;13(1):e1006553. doi: 10.1371/journal.pgen.1006553 (PMC5283763; doi:10.1371/journal.pgen.1006553)

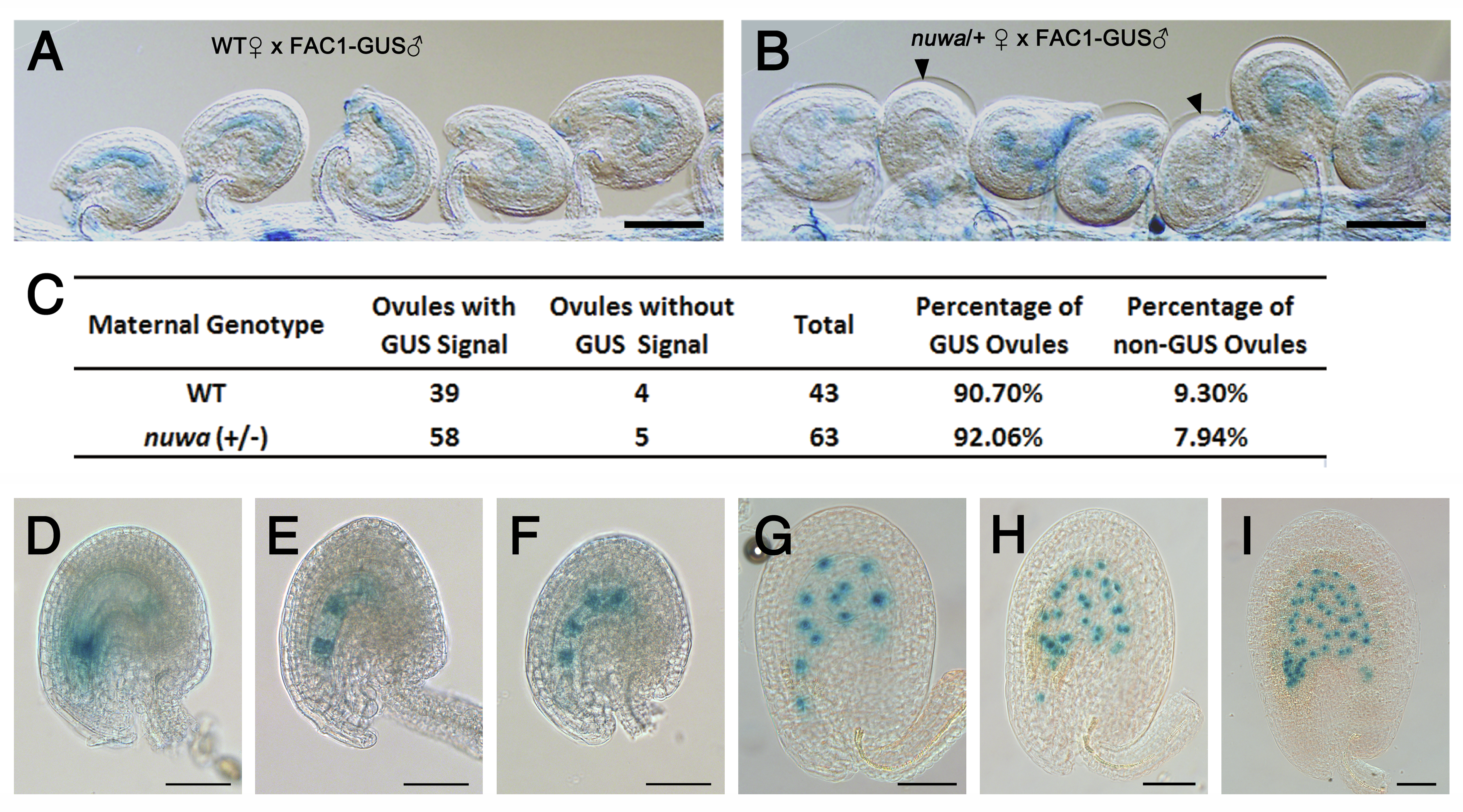

Supplement: S1 Fig — (A and B) Ovules with GUS signal resulting from pollination of emasculated wild type (A) and nuwa-1/+ (B) by pollen of FAC1-GUS marker line. Bars = 100 μm. (C) Statistic analysis of ovules with GUS signal resulting from pollination of emasculated wild type and nuwa-1/+ by pollen of FAC1-GUS marker line. (D-I) FIS2-GUS marker line in wild type background. GUS signals indicate the endosperm nuclei. Bars = 50 μm. (TIF) [file pgen.1006553.s001.tif]

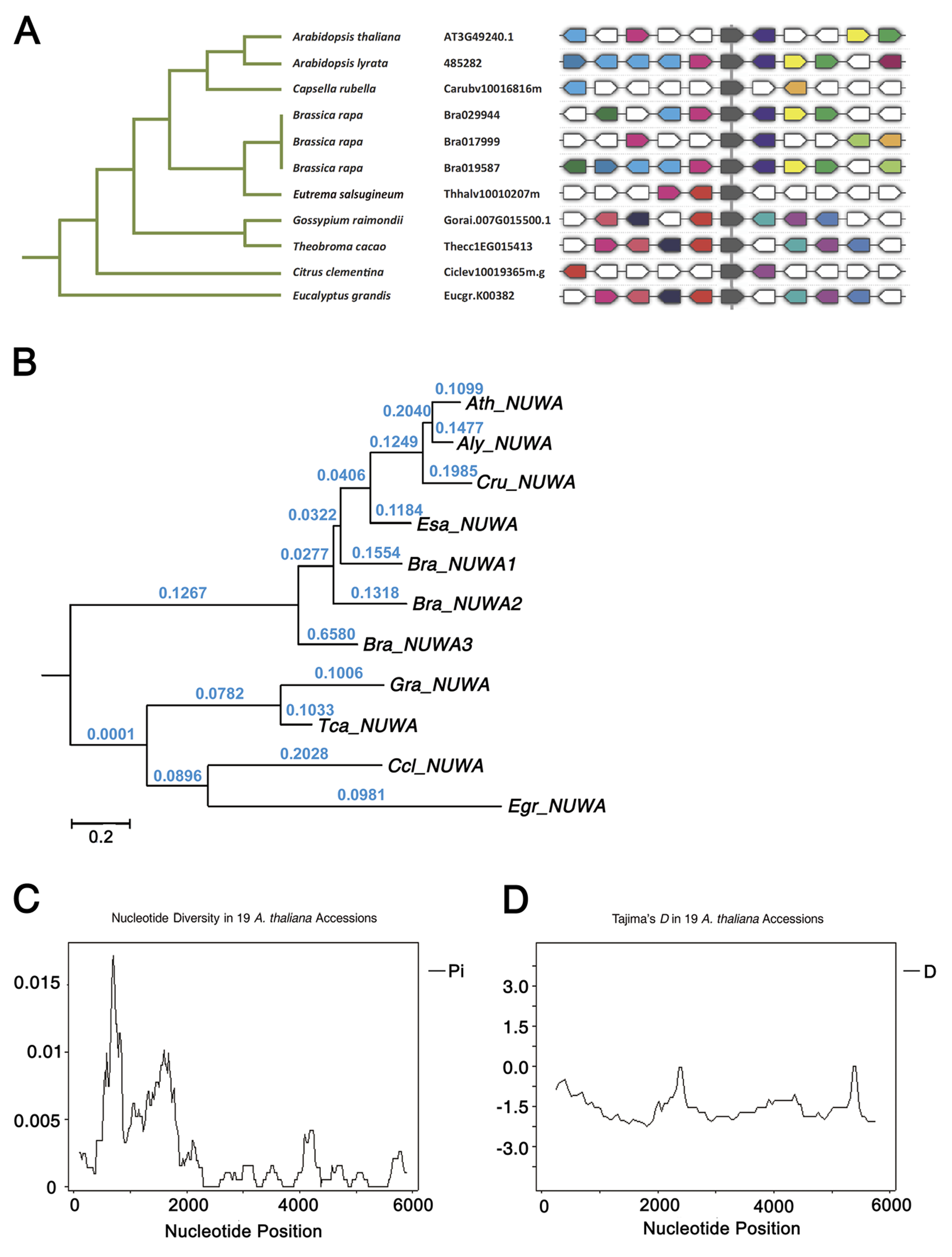

Supplement: S2 Fig — (A) Synteny analysis of NUWA in plant species. Colors of the arrows represent for the various genes in the flanking regions of NUWA. Syntenic orthologs are depicted in the same color, while white arrows are functionally uncharacterized genes. The grey arrows aligned in the middle are NUWA gene and its orthologs in the same direction in each genome. Topology shown here is a species tree based on Phytozome v10. (B) Phylogenetic tree of NUWA and its orthologs. Scale bar, 0.2 amino acid substitutions per site. Ath, Arabidopsis thaliana; Aly, Arabidopsis lyrata; Cru, Capsella rubella; Bra, Brassica rapa; Esa, Eutrema salsugineum; Gra, Gossypium raimondii; Tca, Theobroma cacao; Ccl, Citrus clementina; Egr, Eucalyptus grandis. (C) Nucleotide diversity in the genomic region centered on NUWA among 19 A. thaliana accessions: Col-0, Bur-0, Can-0, Ct-1, Edi-0, Hi-0, Kn-0, Ler-0, Mt-0, No-0, Oy-0, Po-0, Rsch-4, Sf-2, Tsu-0, Wil-2, Ws-0, Wu-0 and Zu-0. (D) Tajima’s D value along the 6 kb genomic region centered on NUWA based on the same set of 19 A. thaliana accessions. (TIF) [file pgen.1006553.s002.tif]

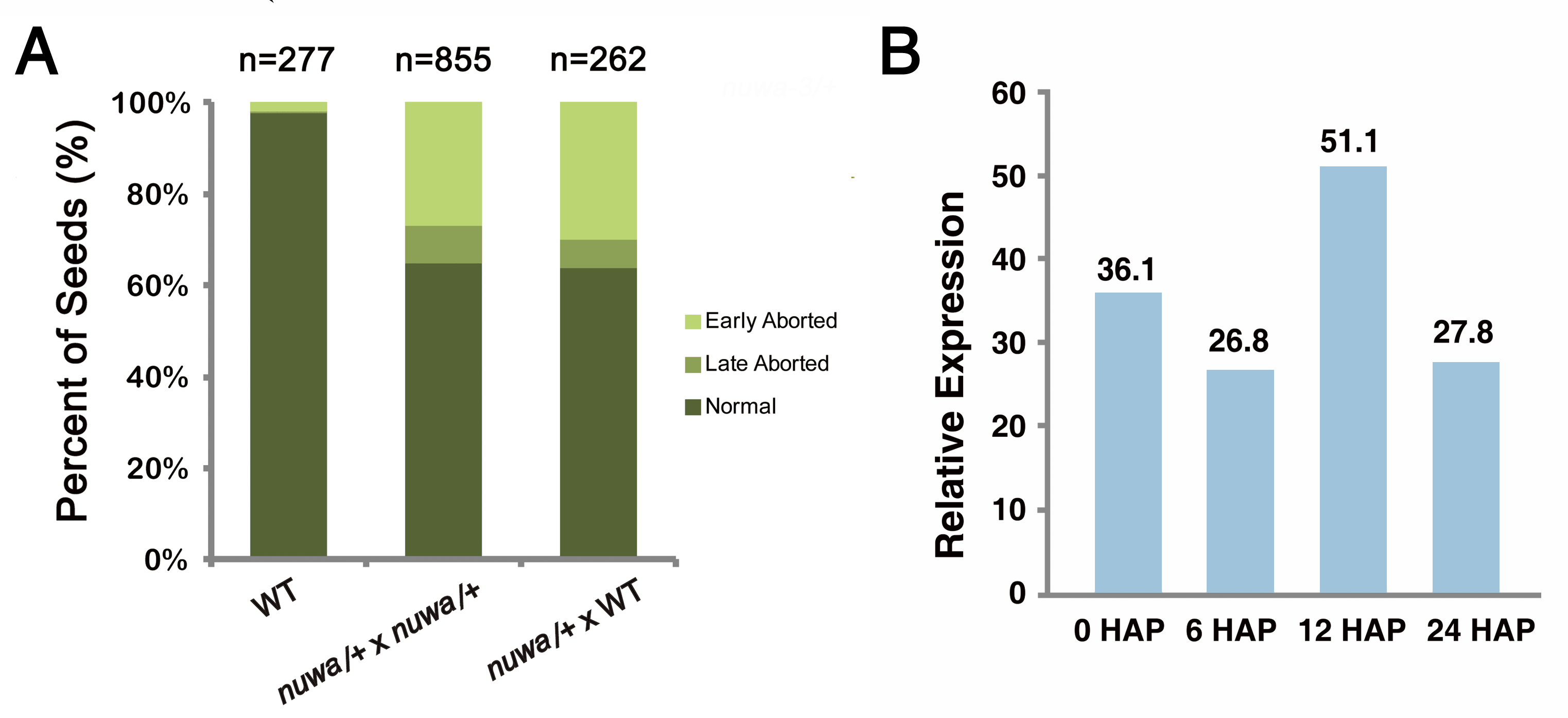

Supplement: S3 Fig — (A) Percentage of seeds in wild type and nuwa-1/+ self crossed siliques and siliques resulted from emasculated nuwa-1/+ pollinated by wild type pollen. (B) RNA-seq data of the transcription level of NUWA in ovules at different developmental stages. (TIF) [file pgen.1006553.s003.tif]

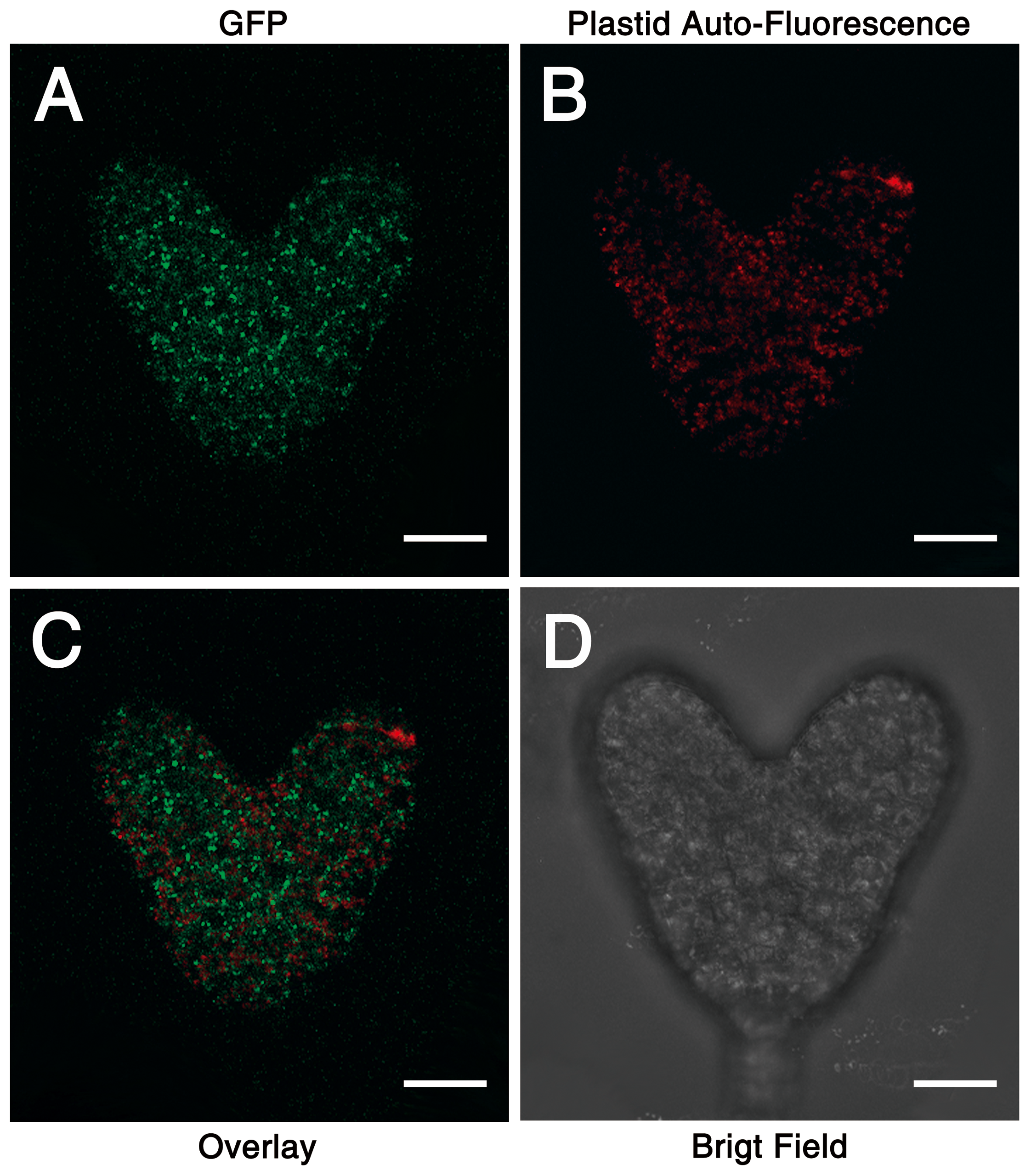

Supplement: S4 Fig — (A-D) GFP signals (A), autofluorescence of chloroplast (B), the overlay of both signals (C) and bright field (D) in heart-stage embryo of pNUWA:NUWA-GFP transgenic plant. Bar = 20 μm. (TIF) [file pgen.1006553.s004.tif]

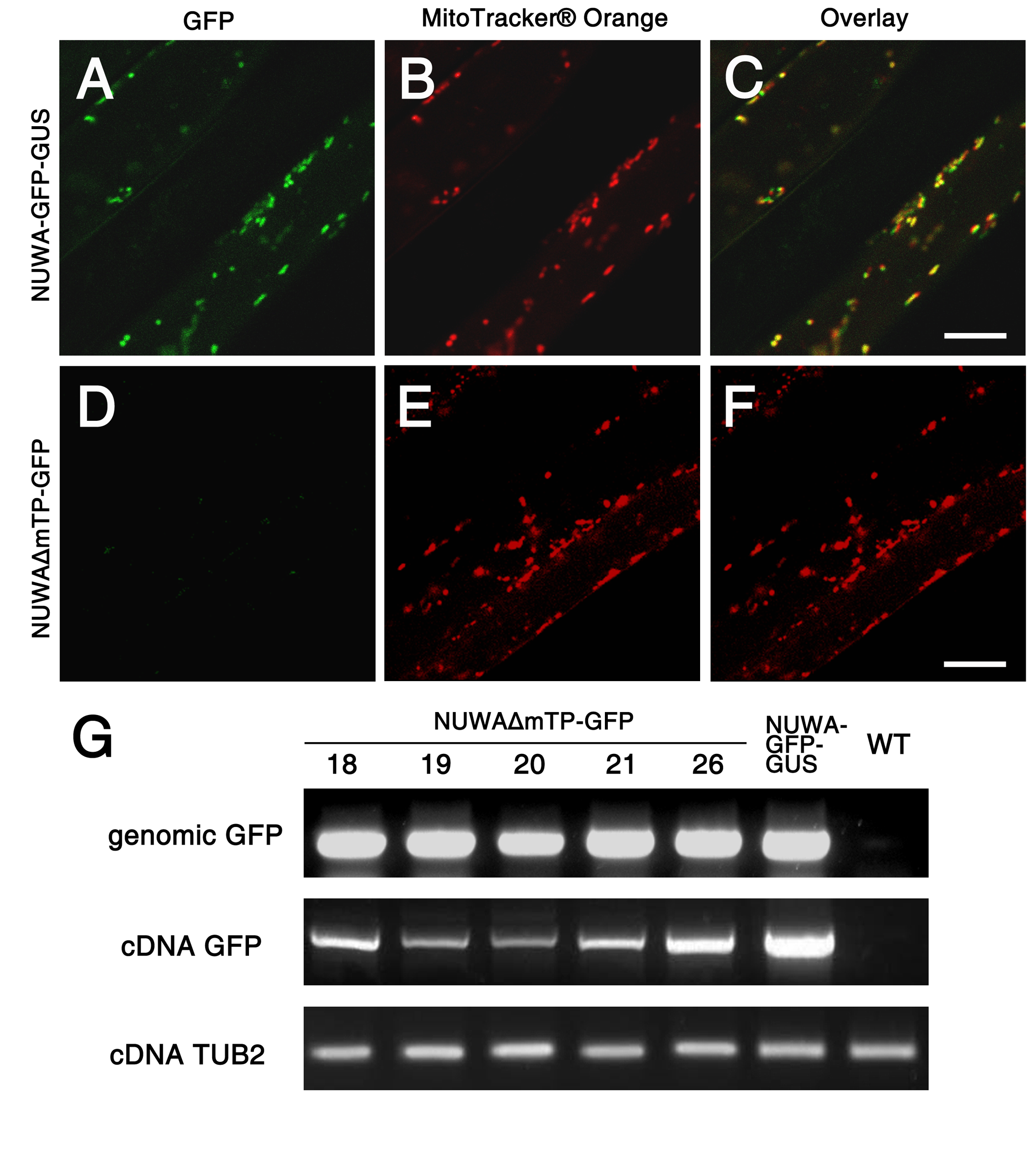

Supplement: S5 Fig — (A-C) GFP signals (A), MitoTracker Orange signals (B) and the overlay of both signals (C) in root cells of pNUWA:NUWA-GFP transgenic seedling. Bar = 5 μm. (D-F) GFP signals (D), MitoTracker Orange signals (E) and the overlay of both signals (F) in root cells of pNUWA:NUWAΔmTP-GFP transgenic seedling. Bar = 5 μm. (G) The expression level of GFP in seedling of five pNUWA:NUWAΔmTP-GFP transgenic lines and one of the pNUWA::NUWA-GFP-GUS transgenic lines. The genomic GFP sequence and the tubulin2 sequences were amplified as controls. (TIF) [file pgen.1006553.s005.tif]

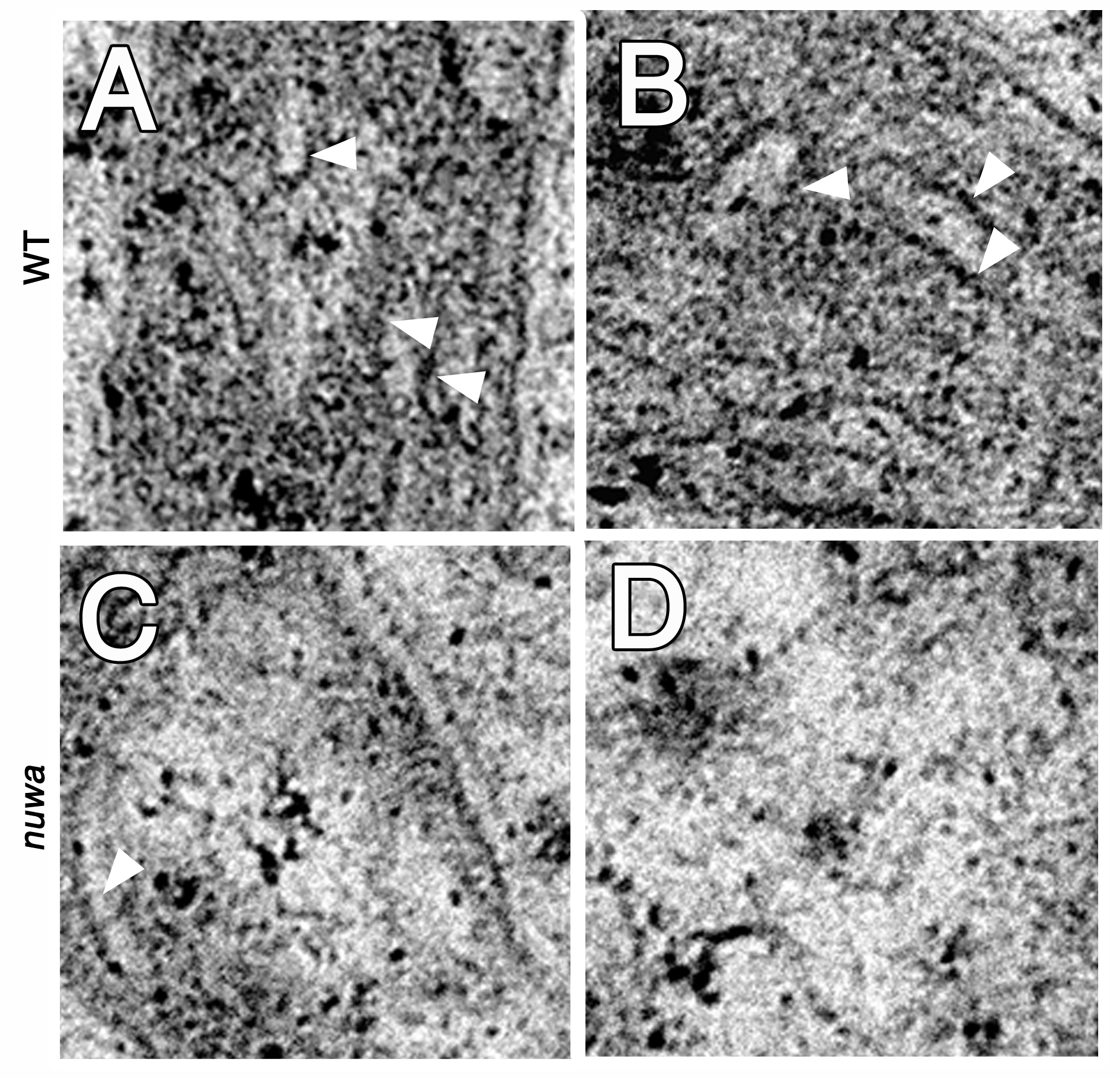

Supplement: S6 Fig — (A) Enlarged part of mitochondria in Fig 5B marked by the white frame. (B) Enlarged part of mitochondria in Fig 5C marked by the white frame. (C) Enlarged part of mitochondria in Fig 5D marked by the white frame. (D) Enlarged part of mitochondria in Fig 5E marked by the white frame. White arrowheads indicate the inner membranes. (TIF) [file pgen.1006553.s006.tif]

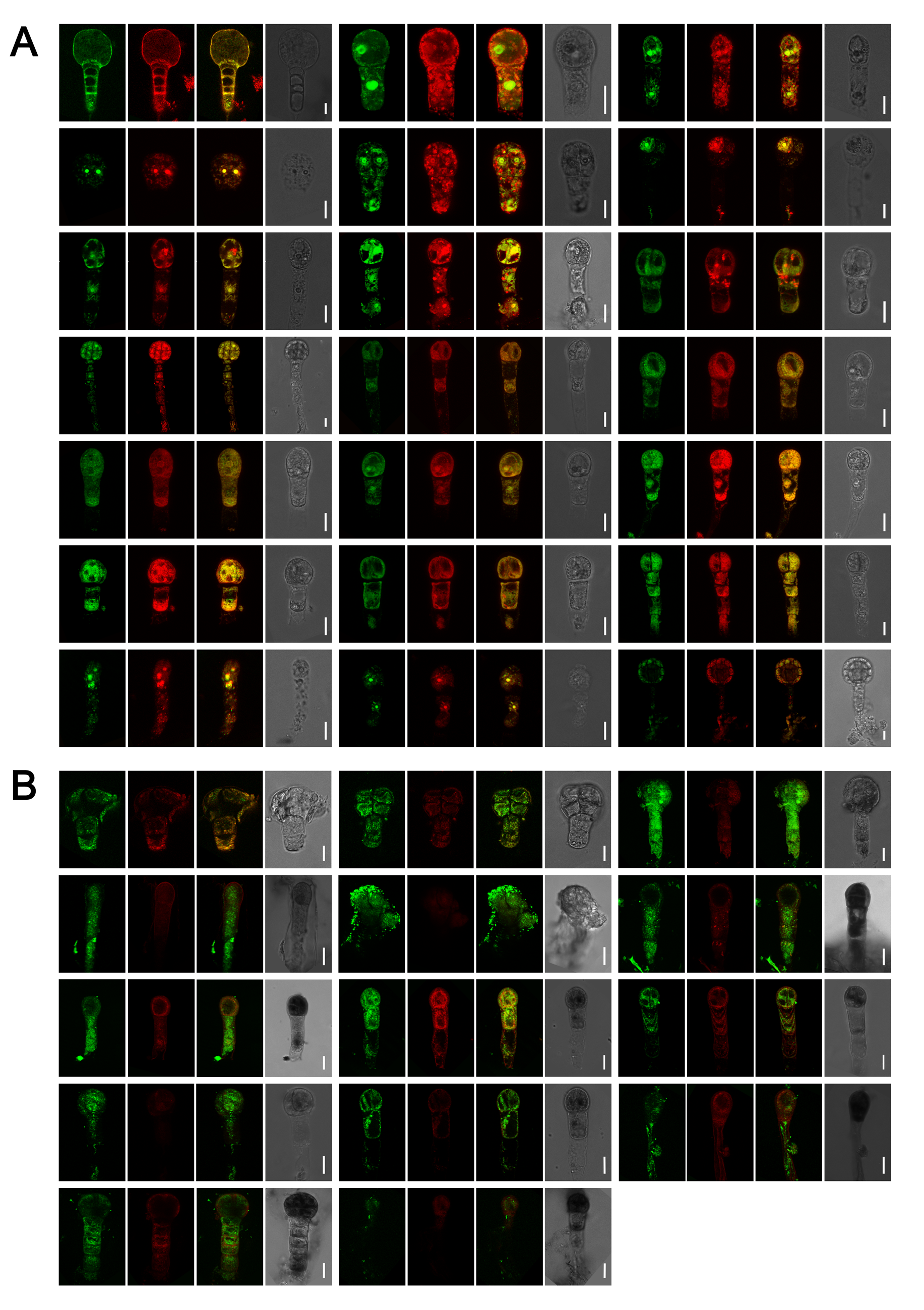

Supplement: S7 Fig — (A-B) JC-1 fluorescence in isolated wild type embryos (A) and nuwa-1/+ mutant embryos (B) at different developmental stages. For each embryo, green fluorescence, red fluorescence, overlay the two fluorescence and bright field are shown. Bars = 10 μm. (TIF) [file pgen.1006553.s007.tif]

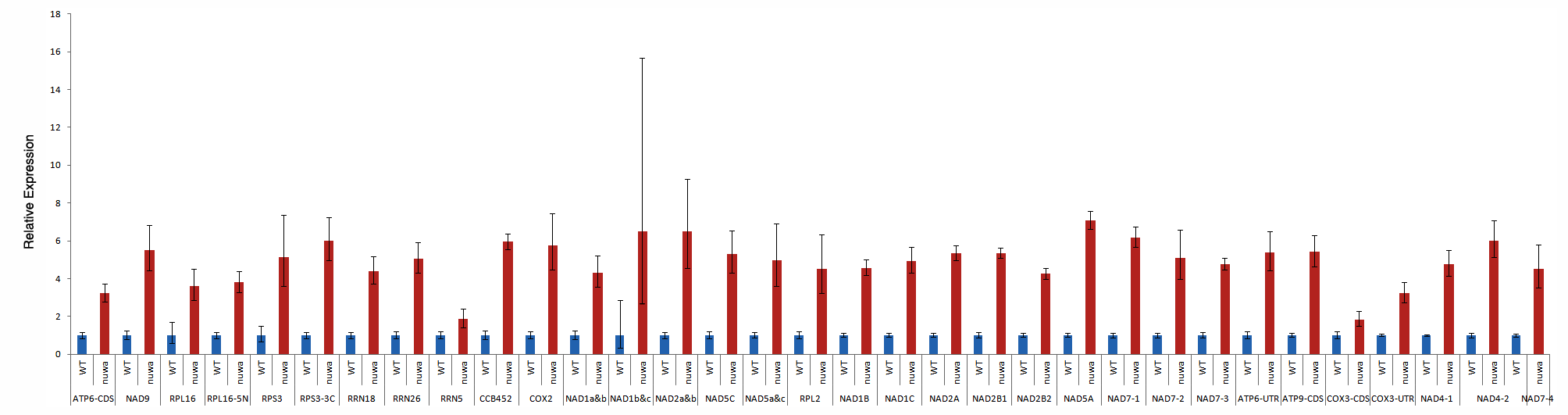

Supplement: S8 Fig — Error bars, mean ± SD. (TIF) [file pgen.1006553.s008.tif]
